# Investigation of the Antitumor Activity and Toxicity of Tumor-Derived Exosomes Fused with Long-Circulating and pH-Sensitive Liposomes Containing Doxorubicin

**DOI:** 10.3390/pharmaceutics14112256

**Published:** 2022-10-22

**Authors:** Eliza Rocha Gomes, Fernanda Rezende Souza, Geovanni Dantas Cassali, Adriano de Paula Sabino, André Luis Branco de Barros, Mônica Cristina Oliveira

**Affiliations:** 1 Department of Pharmaceutical Products, Faculty of Pharmacy, Universidade Federal de Minas Gerais, Av. Antônio Carlos, 6627, Belo Horizonte 31270-901, Minas Gerais, Brazil; 2 Department of General Pathology, Institute of Biological Sciences, Universidade Federal de Minas Gerais, Av. Antônio Carlos, 6627, Belo Horizonte 31270-901, Minas Gerais, Brazil; 3 Department of Clinical and Toxicological Analysis, Faculty of Pharmacy, Universidade Federal de Minas Gerais, Av. Antônio Carlos, 6627, Belo Horizonte 31270-901, Minas Gerais, Brazil

**Keywords:** acute toxicity, breast cancer, metastasis, exosomes, liposomes, doxorubicin

## Abstract

Exosome–liposome hybrid nanocarriers containing chemotherapeutic agents have been developed to enhance drug delivery, improve the efficacy of the treatment of metastatic cancer, and overcome chemoresistance in cancer therapy. Thus, the objectives of this study were to investigate the toxicological profiles of exosomes fused with long-circulating and pH-sensitive liposomes containing doxorubicin (ExoSpHL-DOX) in healthy mice and the antitumor activity of ExoSpHL-DOX in Balb/c female mice bearing 4T1 breast tumors. The acute toxicity was determined by evaluating the mortality and morbidity of the animals and conducting hematological, biochemical, and histopathological analyses after a single intravenous administration of ExoSpHL-DOX. The results of the study indicated that the ExoSpHL-DOX treatment is less toxic than the free doxorubicin (DOX) treatment. ExoSpHL-DOX showed no signs of nephrotoxicity, even at the highest dose of DOX, indicating that the hybrid nanosystem may alter the distribution of DOX and reduce the kidney damage. Regarding the antitumor activity, ExoSpHL-DOX showed an antitumor effect compared to the control group. Furthermore, the hybrid nanocarrier of tumor-derived exosomes fused with long-circulating and pH-sensitive liposomes reduced the number of metastatic foci in the lungs. These results indicate that ExoSpHL-DOX may be a promising nanocarrier for the treatment of breast cancer, reducing toxicity and inhibiting metastasis, mainly in the lungs.

## 1. Introduction

Cancer is one of the leading causes of death and an important barrier to efforts to increase life expectancy across the world. Annually, more than 19 million people develop cancer and approximately 10 million people die from the disease. Breast cancer is the most commonly diagnosed cancer in women, with 2.3 million new cases [1]. Doxorubicin (DOX) is a chemotherapeutic drug used as the first-line treatment for breast cancer. However, this drug causes serious toxic effects, mainly dose-dependent cardiotoxicity, which limits its clinical use [2,3]. To minimize the adverse effects caused by DOX, liposomes have been used in the treatment of patients [2,4]. However, there is no increase in the therapeutic efficacy of liposomal formulations, mainly in DOX-resistant cancer, compared to conventional DOX [2]. In order to improve the therapeutic efficacy of DOX, liposomes can be fused with exosomes released by breast cancer cells. Tetraspanins and integrins, which are present on the surface of exosomes, can facilitate fusion and membrane interactions, achieving a greater cell uptake of fused vesicles [5,6]. Considering the potential strategy of liposome and exosome fusion, Gomes and coworkers [7] developed and characterized a hybrid nanocarrier of tumor-derived exosomes fused with long-circulating and pH-sensitive liposomes containing DOX (ExoSpHL-DOX) for the treatment of breast cancer. The mean diameter of the developed formulation was equal to 100.8 ± 7.8 nm, the polydispersity index (PDI) was 0.122 ± 0.004, and the encapsulated DOX content was equal to 83.5 ± 2.5%. ExoSpHL-DOX was shown to be stable at 4 °C for 60 days. The study of the release of DOX from ExoSpHL-DOX in dilution media with different pH values confirmed the pH sensitivity that is characteristic of the nanosystem, and the cytotoxic study of the 4T1 murine breast cancer cell line demonstrated that the ExoSpHL-DOX treatment significantly reduced the cancer cell viability. Herein, we investigated the toxicological profile of ExoSpHL-DOX in healthy mice and the antitumor activity of ExoSpHL-DOX in Balb/c female mice bearing 4T1 breast tumors.

## 2. Materials and Methods

### 2.1. Chemicals

1,2-Dioleoyl-sn-glycero-3-phosphoethanolamine (DOPE) and 1,2-distearoyl-sn-glycero-3-phosphoethanolamine-N-[amino(polyethyleneglycol)-2000 (DSPE-PEG_2000_) were supplied by Lipoid GmbH (Ludwigshafen, Germany). Cholesterol hemisuccinate (CHEMS), DOX, phosphate-buffered saline (PBS), sodium hydroxide, 4-(2-hydroxyethyl)piperazine-1-ethanesulfonic acid (HEPES), and sodium bicarbonate were obtained from Sigma-Aldrich (St. Louis, MO, USA). Total exosome isolation reagent was obtained from Thermo Fisher Scientific (Waltham, MA, USA).

### 2.2. Cells

The 4T1 murine breast cancer cells were purchased from the American Type Culture Collection (ATCC) (Manassas, VA, USA). Roswell Park Memorial Institute (RPMI) 1640 Medium and fetal bovine serum (FBS) were obtained from Gibco Life Technologies (Carlsbad, CA, USA). Trypsin was obtained from Sigma-Aldrich (St. Louis, MO, USA). A mycoplasma test using Hoechst fluorescence staining was performed on the cell line.

### 2.3. Isolation of Exosomes

The 4T1 cells were grown in RPMI-1640 supplemented with 10% of ultracentrifuged FBS and maintained at 37 °C and 5% CO_2_ in a humidified atmosphere. When the 4T1 cells reached an approximate confluence of 80%, the supernatant was removed from the cell culture flask T-75. The exosome isolation reagent was added to the supernatant (1:2 *v*/*v* ratio, respectively) and kept in a refrigerator for 15 h. After that time, the mixture was centrifuged at 10,000× *g* for 1 h at 4 °C using a centrifuge from Thermo Scientific, model Heraeus Multifuge X 1R. The pellet was dissolved in a mixture of chloroform and methanol (1:1 *v*/*v* ratio).

### 2.4. Preparation of ExoSpHL-DOX

ExoSpHL-DOX was prepared using the Bangham method [8] followed by extrusion for the size calibration. Chloroform aliquots of DOPE, CHEMS, and DSPE-PEG_2000_ (5.7:3.8:0.5 molar ratio, respectively) and exosomes in a mixture of chloroform and methanol were added to a round-bottomed flask to obtain a lipid film. For each mL of liposome, we added an exosome pellet obtained from 2 mL of cell supernatant (concentration of 3.6 × 10^10^ particles/mL). After the evaporation of the solvents, NaOH 0.228 M solution was added to ionize the CHEMS molecules and, subsequently, promote the formation of vesicles. The hydration of the lipid film was carried out under agitation with an ammonium sulfate solution (300 mM, pH 7.4). The vesicles obtained were calibrated by extrusion using the Lipex Biomembranes extruder, Model T001 (Vancouver, BC, Canada) [9]. The external ammonium sulfate was removed by ultracentrifugation (Ultracentrifuge Optima^®^ L-80XP, Beckman Coulter, Brea, CA, USA) at 150,000× *g*, 4 °C, for 120 min. The pellet was resuspended with HEPES buffered saline (HBS). The vesicles were incubated with a DOX solution for 2 h in the dark at room temperature. The non-encapsulated DOX was removed by ultracentrifugation using the same method as that described above. The final pellet was resuspended with HBS. Blank breast-tumor-derived exosomes fused with long-circulating and pH-sensitive liposomes (ExoSpHL) and long-circulating and pH-sensitive liposomes containing DOX (SpHL-DOX) were prepared in the same way without the addition of DOX and exosomes, respectively.

### 2.5. ExoSpHL-DOX Characterization

#### 2.5.1. Determination of the Diameter, Polydispersity Index, and Zeta Potential

The mean diameter and the polydispersity index (PDI) of ExoSpHL-DOX were measured by dynamic light scattering (DLS). The zeta potential value was determined by DLS combined with electrophoretic mobility. To perform both analyses, 50 µL of ExoSpHL-DOX was diluted in 1 mL of HBS, and the Zetasizer Nano ZS90 equipment was used (Malvern Instruments Ltd., Worcestershire, UK).

#### 2.5.2. Determination of the Content of DOX

The DOX content was measured by high-performance liquid chromatography (HPLC). The mobile phase consisted of methanol:phosphate buffer pH 3.0 (65:35 *v*/*v*). Samples were injected (20 μL), and the separation was performed with an ACE^®^ C8 column, 25 cm × 4.6 mm, 5 μm (Merck, Darmstadt, Germany), at a flow rate of 1.0 mL/min. The detection was performed in the model 2475 fluorescence mode (Waters Instruments, Milford, MA, USA), with excitation and emission wavelengths of 470 nm and 555 nm, respectively [10]. The ExoSpHL-DOX was opened with isopropyl alcohol (1:2 *v*/*v*, respectively) and diluted in the mobile phase. The encapsulation percentage (EP) of DOX in ExoSpHL-DOX was calculated according to the following equation:

$$\mathrm{DOX}\;\mathrm{encapsulation}\;\mathrm{percentage}\;\left(\%\right)=\frac{\left[\mathrm{DOX}\right]\;\mathrm{in}\;\mathrm{purified}\;\mathrm{vesicles}}{\left[\mathrm{DOX}\right]\;\mathrm{in}\;\mathrm{non}-\mathrm{purified}\;\mathrm{vesicles}}\times 100$$

### 2.6. Animals

Healthy female Balb/c mice aged 8–10 weeks and approximately 18 g in weight were obtained from Central Biotery, Universidade Federal de Minas Gerais (UFMG, Belo Horizonte, Brazil). The mice were kept in plastic cages with free access to food and water and under standardized light/dark cycle conditions. All protocols were approved by the Ethics Committee for Animal Experiments from the Universidade Federal de Minas Gerais (CEUA/UFMG—protocol number 265/2019).

### 2.7. Acute Toxicity

**Figure A1:**
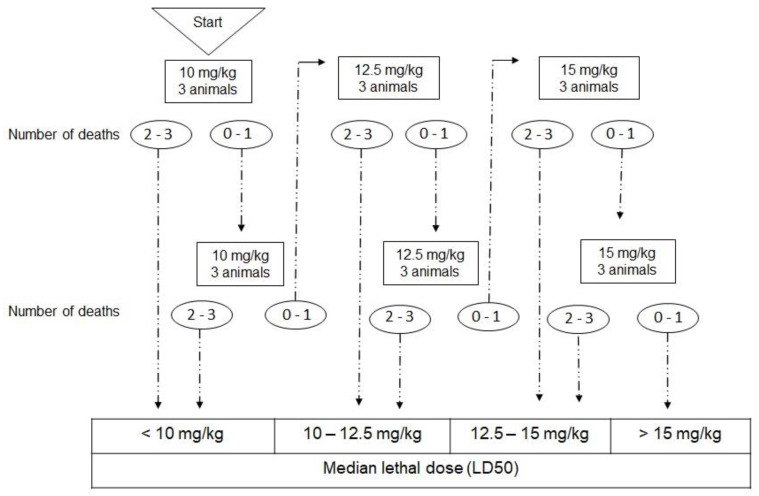
Treatment scheme used to assess the median lethal dose (LD50) after treatment with free DOX.

**Figure A2:**
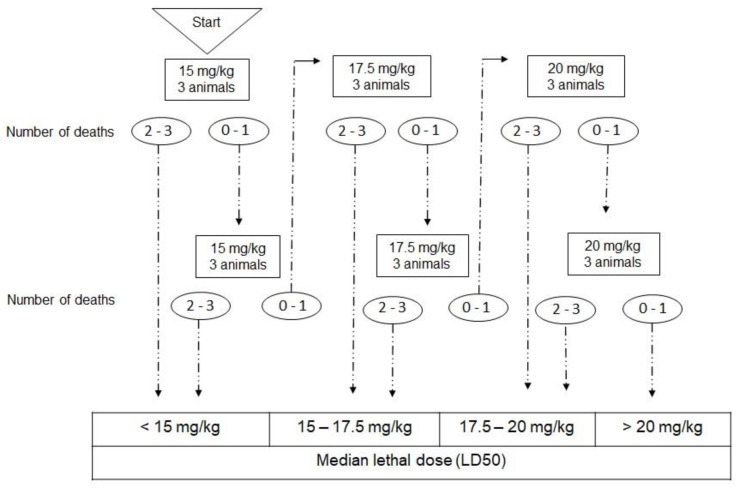
Treatment scheme used to assess the median lethal dose (LD50) after treatment with SpHL-DOX or ExoSpHL-DOX.

The acute toxicity was assessed according to the recommendations of the Organization for Economic Cooperation and Development (OECD) 423 [11], adapted for intravenous administration, as previously performed by our research group [12]. The animals were divided into five groups. Each group intravenously received a single dose of HBS, ExoSpHL, free DOX, SpHL-DOX, or ExoSpHL-DOX. The mice were observed for 14 days in terms of their behavior, weight, and mortality. After the observation period, the animals were intraperitoneally anesthetized with a mixture of xylazine (15 mg/kg) and ketamine (80 mg/kg). The blood was collected by puncture of the brachial plexus for hematological and biochemical analyses. The organs were collected for histopathological analyses. In previous studies, our research group evaluated the toxicity of 10 mg/kg and 15 mg/kg of free DOX and SpHL-DOX in mice. A weight loss of around 5%, prostration, and intense piloerection were observed in the animals treated with free DOX (15 mg/kg). No significant signs of toxicity were observed in the animals treated with SpHL-DOX (15 mg/kg). Based on the findings of this study, the initial doses proposed in this study were 10 mg/kg of free DOX and 15 mg/kg of SpHL-DOX and ExoSpHL-DOX [12]. According to the OECD guideline [11], initially, each treatment group was composed of 3 animals. If the dose tested was capable of causing the death of 2 or more animals in the group, the dosing of 3 additional animals at the previous lowest dose level was required. However, if the tested dose was able to cause one or no death, the next step was the dosing of 3 additional animals with the same dose. In the case of the confirmation of the results of one or no death, it was necessary to administer the following higher dose level to 3 additional animals. The dose scheme used to assess the median lethal dose (LD50) after the treatments with the free DOX and formulations (SpHL-DOX and ExoSpHL-DOX) is presented in Appendix A (Figure A1 and Figure A2, respectively). LD50 refers to the single dose of DOX that was required to cause death in 50 percent of the animals tested.

#### 2.7.1. Hematology and Biochemistry Analyses

For the hematological analysis, the blood was collected in tubes containing anticoagulant (EDTA 0.1 M) and inserted into the automated hematological analyzer HEMOVET 2300 (Hemovet, São Paulo, Brazil). Hematological parameters related to red and white blood cells were evaluated for each treatment group. For the biochemical analysis, the blood was centrifuged (3000 rpm, 15 min), and the plasma obtained was collected. The tests were performed with the Bioplus BIO-2000 semiautomatic analyzer (Bioplus, São Paulo, Brazil) using commercial kits (Labtest, Lagoa Santa, Brazil). The renal, liver, and cardiac functions were evaluated for each treatment group.

#### 2.7.2. Histopathological Analysis

The liver, kidneys, spleen, and heart were harvested and fixed in formalin (10% *w*/*v* in phosphate-buffered saline (PBS), pH 7.4) and incorporated in paraffin blocks. Consecutive histological sections were prepared and stained by the hematoxylin and eosin routine method. The slides were evaluated by trained pathologists, and images of histological sections were captured using a digital camera connected to an optical microscope, Olympus BX-40 (Olympus, Tokyo, Japan).

### 2.8. Evaluation of the Antitumor Activity

The 4T1 breast cancer cells were injected into the right flank of the female Balb/c mice (1.0 × 10^6^ cells in 100 µL PBS). When the tumor volume reached approximately 100 mm^3^, the animals were randomly divided into five treatment groups, each containing six animals. Each group intravenously received five administrations of HBS, ExoSpHL, free DOX, SpHL-DOX, or ExoSpHL-DOX. The cumulative dose of DOX was 25 mg/kg. The dose used in this study was based on a previous study of our research group [13]. The antitumor activity was evaluated based on the tumor volume (TV), calculated as previously described [14], where TV = 0.52 × (d1 × d2^2^), d1 and d2 being the largest and the smallest perpendicular diameters, respectively. Six measurements of the diameters of the tumors were carried out during ten days of treatment using a caliper MIP/E-103 (Mitutoyo, Suzano, São Paulo, Brazil). The TV at day 0 was considered as 100%, and changes in the TV were determined every two days by calculating the percentages of the TV increase or decrease. The relative tumor volume (RTV) and inhibition percentage of tumor growth (TGI) were calculated according to the following equations:

$$\mathrm{RTV}=\frac{\mathrm{TV}\;\mathrm{on}\;\mathrm{day}\;10}{\mathrm{TV}\;\mathrm{on}\;\mathrm{day}\;0}$$

$$\mathrm{TGI}=1-\frac{\;\mathrm{RTV}\;\mathrm{of}\;\mathrm{each}\;\mathrm{treatment}}{\mathrm{RTV}\;\mathrm{of}\;\mathrm{the}\;\mathrm{control}\;\mathrm{group}}\times 100$$

On day 10, the animals were intraperitoneally anesthetized with a mixture of xylazine (15 mg/kg) and ketamine (80 mg/kg). The liver, spleen, lungs, heart, and tumor were collected for the histopathological analysis.

### 2.9. Statistical Analyses

To confirm the normality and homoscedasticity of variance, D’Agostino and Shapiro–Wilk tests were applied, respectively. The differences between the experimental groups were tested by analysis of variance (one-way ANOVA followed by Tukey’s test). If the data were not normal or homoscedastic, the Kruskal–Wallis test with Dunn’s post-test was used for the same purpose. The two-way ANOVA test with Tukey’s post-test was also used to relate two different independent variables with respect to one dependent variable. Values of *p* < 0.05 were considered significant. The analyses were performed using the GraphPad Prism software (version 6.00, La Jolla, CA, USA).

## 3. Results

### 3.1. ExoSpHL-DOX Characterization

ExoSpHL-DOX presented a mean diameter of 105.4 ± 2.9 nm and a PDI value of 0.132 ± 0.010, indicating the presence of monodisperse vesicles. The zeta potential value was near neutrality (−6.4 ± 1.2 mV), as expected for vesicles that contain PEG in their composition. The encapsulation percentage of DOX was 88.5 ± 2.4%, achieved by using the ammonium sulfate gradient method.

### 3.2. Acute Toxicity Study

#### 3.2.1. Evaluation of Animal Mortality and Morbidity

The HBS and ExoSpHL treatments did not show significant differences in relation to the mortality and morbidity. These findings indicate the lack of toxicity of the treatments applied to the mice in the control groups. The LD50 assessment of animals treated with free DOX started with 10 mg/kg and with 15 mg/kg for both formulations (SpHL-DOX and ExoSpHL-DOX). For the free DOX treatment, the 10 mg/kg dose showed no significant signs of toxicity in the first three animals tested. Thus, the next step was the dosing of three additional animals with the same dose. The results remained the same in all animals; therefore, the following higher dose level was injected into three animals. After 8 days of application of 12.5 mg/kg of DOX, piloerection and ascites in the animals were observed. It is worth mentioning that the most notable result was a weight loss of 13%, observed at day 12 post-administration. However, no deaths were observed. Therefore, the next step was the dosing of three additional animals with the same dose. The previous observations were confirmed, and there was one death on day 13. According to Appendix A (Figure A1), the next step was the dosing of three additional animals at the following higher dose level (15 mg/kg). For the first three mice evaluated, there was one death on day 10 and one death on day 12 of the study. In addition, intense piloerection was observed in all animals. Additionally, we observed a loss of weight in the animals ranging between 7% and 20% during the days after treatment. Therefore, according to the OECD 423 guideline [11], the LD50 value for free DOX treatment is between 12.5 and 15 mg/kg for this experimental model. Thus, 15 mg/kg was the last dose tested for the treatment with the free drug.

The studies carried out using the SpHL-DOX and ExoSpHL-DOX treatments started with a dose of 15 mg/kg. For both treatments, there was no death or weight loss in all of the six animals tested. After treatment with a dose of 17.5 mg/kg, no deaths were observed. However, there was a 5% weight loss in the animals. According to these results, we repeated the experiments for the groups treated with SpHL-DOX and ExoSpHL-DOX at a dose of 17.5, using three more animals per treatment, and the previous observations were confirmed. We followed the treatment scheme (Appendix A, Figure A2), increasing the dose to 20 mg/kg for the SpHL-DOX and ExoSpHL-DOX treatments. After treatments with the 20 mg/kg dose, we observed two deaths on day 6 and one death on day 8 in the SpHL-DOX treatment group. In the ExoSpHL-DOX treatment group, one death on day 6, one death on day 8, and one death on day 10 occurred. In addition, during the days after treatment, the animals treated with ExoSpHL-DOX and SpHL-DOX had weight losses of 10% and 20%, respectively. Therefore, according to the OECD 423 guideline [11], the LD50 value for the SpHL-DOX and ExoSpHL-DOX treatments is between 17.5 and 20 mg/kg for this experimental model. Thus, 20 mg/kg was the last dose tested for both formulations.

#### 3.2.2. Hematological Analysis

The hematological parameters of the mice treated with DOX, SpHL-DOX, and ExoSpHL-DOX are shown in Table 1. The HBS and ExoSpHL treatment groups showed no significant difference; therefore, only the HBS treatment group was expressed as a control. The evaluation of white blood cells (WBC) showed that there was an increase in the WBC count after treatment with DOX at a dose of 12.5 mg/kg when compared to the control group (HBS). The other treatments did not change the WBC count when compared to HBS. The same increase was observed in the granulocytes (neutrophils, eosinophils and basophils) and agranulocytes (lymphocytes and monocytes) in the group treated with DOX at a dose of 12.5 mg/kg when compared to HBS. The other treatments did not change the granulocyte and agranulocyte count when compared to HBS. Regarding the red blood cells, the number of red blood cells (RBC), amount of hemoglobin (HGB), and hematocrit (HCT) showed a decrease in the mice treated with DOX at a dose of 12.5 mg/kg and did not change among the other treatments when compared to the control. The platelet count showed no difference between all the treatments.

#### 3.2.3. Biochemical Analysis

The biochemical parameters of the mice treated with DOX, SpHL-DOX, and ExoSpHL-DOX are shown in Table 2. The HBS and ExoSpHL treatment groups showed no significant difference; therefore, only the HBS treatment group was expressed as a control. The renal function was evaluated by measuring the creatinine and urea. There was no change in the creatinine values in all treatments when compared to the control. For the quantification of the urea, only the DOX treatment at a dose of 12.5 mg/kg presented an increase in relation to the control group. The hepatic function was evaluated by determining the alanine aminotransferase (ALT) and aspartate aminotransferase (AST) activity, and the doses used did not cause liver damage, since there was no significant difference in the serum levels of ALT and AST in all the treatment groups when compared to the control group. Cardiac injury was assessed by measuring the creatine kinase-MB (CK-MB) activity. The DOX treatment at a dose of 12.5 mg/kg and SpHL-DOX and ExoSpHL-DOX treatments at a dose of 17.5 mg/kg showed high levels of CK-MB in relation to the control group. Furthermore, the increase in the CK-MB level due to DOX treatment at a dose of 12.5 mg/kg was greater than that of SpHL-DOX and ExoSpHL-DOX at a dose of 17.5 mg/kg.

#### 3.2.4. Histological Analysis

**Figure 1:**
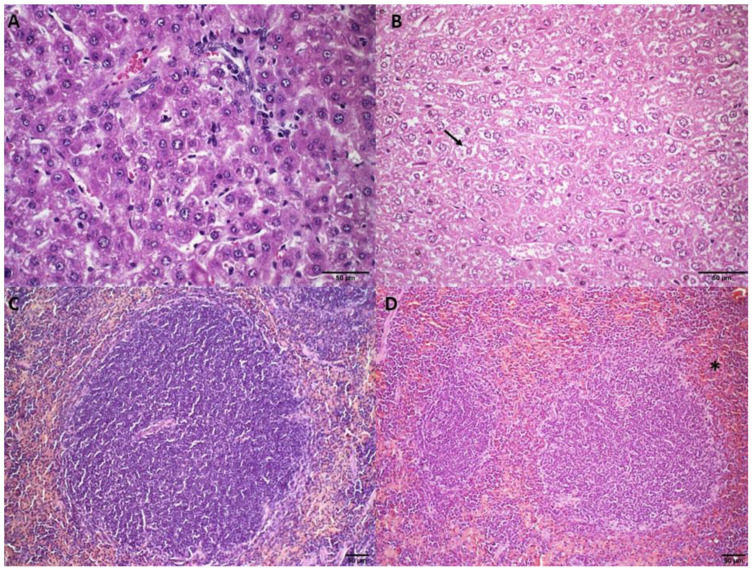
Histological sections of female Balb/c mice liver (**A**,**B**) and spleen (**C**,**D**). (**A**) Represents the control group (HBS) and the groups treated with SpHL-DOX and ExoSpHL-DOX at a dose of 15 mg/kg. (**B**) Represents the groups treated with both doses of free DOX (10 and 12.5 mg/kg) and the groups treated with SpHL-DOX and ExoSpHL-DOX at a dose of 17.5 mg/kg. The arrow indicates the regions of diffuse hydropic degeneration. (**C**) Represents the control group (HBS) and the groups treated with both tested doses of SpHL-DOX and ExoSpHL-DOX (15 and 17.5 mg/kg). (**D**) Represents the groups treated with both tested doses of free DOX (10 and 12.5 mg/kg). The asterisk indicates the increase in erythrocytes in the red pulp. HE, scale bar = 50 µm.

Histological analyses of the different organs were performed at the end of the treatment period. The mice treated with HBS and ExoSpHL presented with the same histopathological profile and, therefore, only the HBS treatment group’s photomicrography was presented as the control group. The liver analysis showed no changes in the SpHL-DOX (15 mg/kg) and ExoSpHL-DOX (15 mg/kg) treatment groups compared to the control group (Figure 1A). In contrast, diffuse hydropic degeneration was observed in the animals treated with DOX (10 and 12.5 mg/kg), SpHL-DOX (17.5 mg/kg), and ExoSpHL-DOX (17.5 mg/kg) (Figure 1B). In the splenic analysis, no changes were observed after the treatments with both doses of SpHL-DOX and ExoSpHL-DOX (15 and 17.5 mg/kg) (Figure 1C). In the animals treated with DOX (10 and 12.5 mg/kg), we observed splenic congestion. The red pulp sinusoids had a large number of erythrocytes (Figure 1D).

**Figure 2:**
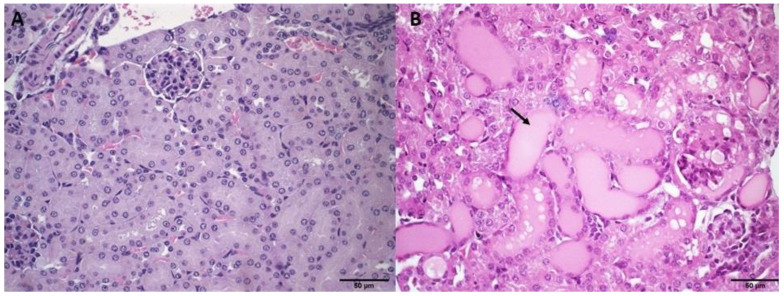
Histological sections of female Balb/c mice kidney. (**A**) Represents the control group (HBS) and the groups treated with both doses of ExoSpHL-DOX (15 and 17.5 mg/kg) and SpHL-DOX at a dose of 15 mg/kg. (**B**) Represents the groups treated with both tested doses of free DOX (10 and 12.5 mg/kg) and SpHL-DOX at a dose of 17.5 mg/kg. The arrow indicates the regions of tubule dilation. HE, scale bar = 50 µm.

The histopathological analysis of the kidneys revealed no changes in the case of the SpHL-DOX (15 mg/kg) and ExoSpHL-DOX (15 and 17.5 mg/kg) treatment groups compared to the control group (Figure 2A). Animals treated with DOX (10 and 12.5 mg/kg) and SpHL-DOX (17.5 mg/kg) presented with tubule dilation and hyalinization of the glomeruli (Figure 2B).

**Figure 3:**
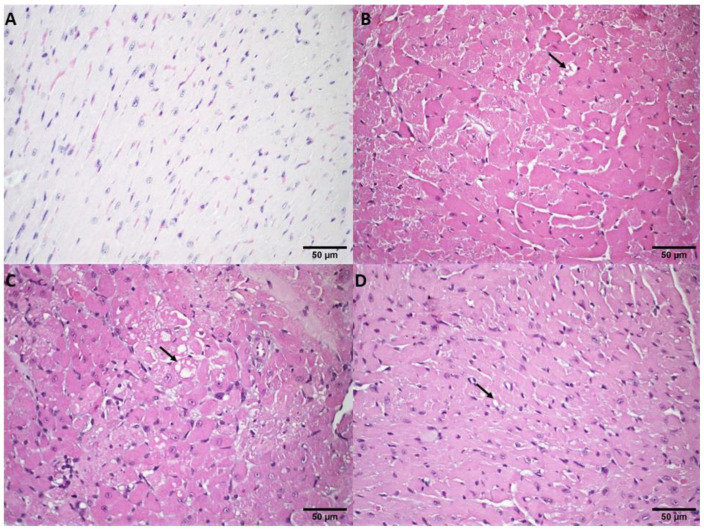
Histological sections of female Balb/c mice heart. (**A**) Represents the control group (HBS). (**B**) Represents the group treated with free DOX at a dose of 10 mg/kg. The arrow indicates the areas of cardiomyocyte vacuolization. (**C**) Represents the group treated with free DOX at a dose of 12.5 mg/kg. The arrow indicates the areas of cardiomyocyte vacuolization that were more intense than (**B**). (**D**) Represents the groups treated with both doses of SpHL-DOX and ExoSpHL-DOX. The arrow indicates the areas of discrete cardiomyocyte vacuolization. HE, scale bar = 50 µm.

The cardiac muscle analysis revealed that, after the DOX treatments, areas of cardiomyocyte vacuolization were observable. Compared to the control group (Figure 3A), the treatment with free DOX (10 and 12.5 mg/kg) (Figure 3B,C, respectively) presented multifocal areas of cardiomyocyte vacuolization. The extent of the lesions was greater in the DOX treatment group at a dose of 12.5 mg/kg. For the SpHL-DOX (15 and 17.5 mg/kg) and ExoSpHL-DOX (15 and 17.5 mg/kg) treatment groups, discrete foci of cardiomyocyte vacuolization were observed (Figure 3D). The analyzed histological sections of all the groups and organs analyzed that showed some difference compared to the control are presented in Appendix B.

### 3.3. Antitumor Activity Evaluation

**Figure 4:**
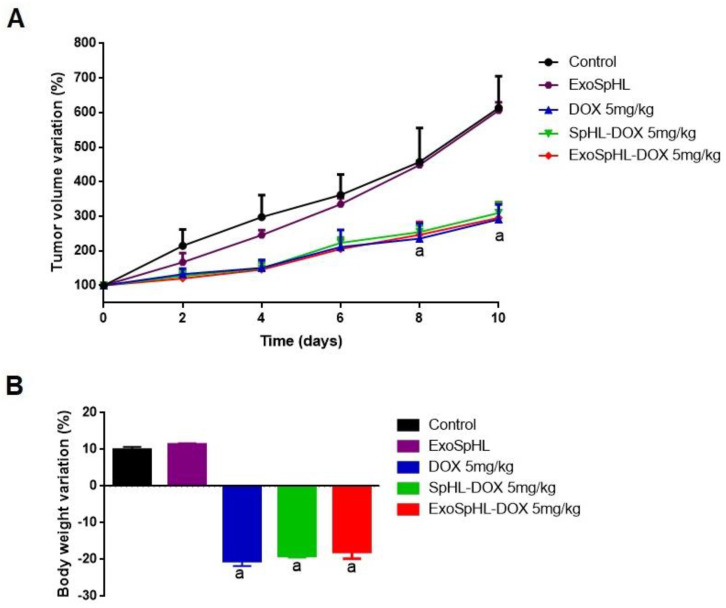
Antitumor efficacy evaluation of female Balb/c mice with 4T1 breast tumors. (**A**) Variation in the mice breast cancer tumor volume after treatment with HBS, ExoSpHL, DOX, SpHL-DOX, and ExoSpHL-DOX. Every 2 days, the animals intravenously received five administrations of HBS (control), ExoSpHL, or DOX at a cumulative dose of 25 mg/kg, SpHL-DOX at a cumulative dose of 25 mg/kg, or ExoSpHL-DOX at a cumulative dose of 25 mg/kg. (**B**) Percentage of body weight variation on day 10 after the administration of HBS (control), ExoSpHL, or DOX at a cumulative dose of 25 mg/kg, SpHL-DOX at a cumulative dose of 25 mg/kg, or ExoSpHL-DOX at a cumulative dose of 25 mg/kg. All data are presented as mean ± SEM, *n* = 6. ^a^ Statistical significance compared to the control group (HBS) and ExoSpHL (*p* < 0.05). Abbreviations: HBS: HEPES buffered saline; ExoSpHL: tumor-derived exosomes fused with long-circulating and pH-sensitive liposomes; DOX: doxorubicin; SpHL-DOX: long-circulating and pH-sensitive liposomes containing doxorubicin; ExoSpHL-DOX: tumor-derived exosomes fused with long-circulating and pH-sensitive liposomes containing doxorubicin; SEM: standard error of the mean.

The antitumor efficacy of the free DOX, SpHL-DOX, and ExoSpHL-DOX treatments was evaluated in the female Balb/c mice with 4T1 breast tumors by assessing the tumor volume variation over time. The tumor volume in the HBS (control) and ExoSpHL treatment groups increased rapidly over time and showed no significant differences between them. By contrast, significant differences in the tumor volume were observed between the control treatment groups and the groups treated with DOX, SpHL-DOX, and ExoSpHL-DOX at a dose of 5.0 mg/kg (Figure 4A). The tumor volume data were confirmed by the RTV values (Table 3). The treatments with formulations containing DOX significantly decreased the tumor growth compared to the control group. However, there was no significant difference between the DOX, SpHL-DOX, and ExoSpHL-DOX treatments at the cumulative dose of 25.0 mg/kg. In addition, the treatments showed similar TGIs, which were close to 50%, compared to the control group.

#### 3.3.1. Evaluation of Body Weight Loss

The body weight loss after the treatments with DOX, SpHL-DOX, and ExoSpHL-DOX was evaluated in the Balb/c female mice with 4T1 breast tumors. The results are presented in Figure 4B. The mice treated with HBS (control) and ExoSpHL presented with similar body weight gain. By contrast, significant differences in the animals’ weights were observed between the control treatments and treatments with DOX, SpHL-DOX, and ExoSpHL-DOX. However, there was no significant difference in body weight loss among the animals treated with DOX, SpHL-DOX, and ExoSpHL-DOX at the cumulative dose of 25.0 mg/kg.

#### 3.3.2. Histological Analysis

**Figure 5:**
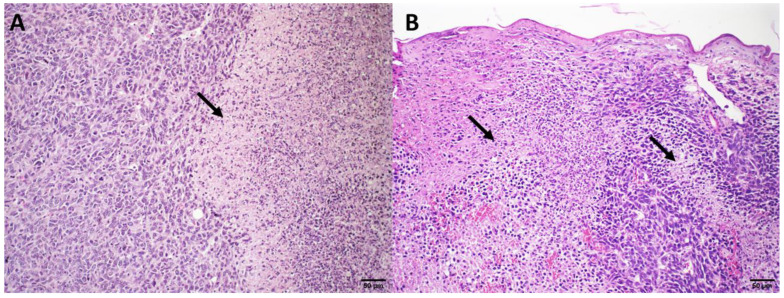
Representative photomicrographs of histological sections of the primary tumors of female Balb/c mice bearing 4T1 breast tumors treated with (**A**) HBS and ExoSpHL. The arrow indicates the central tumor necrosis area. (**B**) DOX, SpHL-DOX, and ExoSpHL-DOX at a cumulative dose of 25 mg/kg. The arrows indicate multiple tumor necrosis areas. HE, scale bar = 50 µm.

Histological analyses of the tumors and different organs were performed at the end of the treatment period. The 4T1 tumor cells grow in a solid arrangement. The proliferation of pleomorphic cells and a high mitotic index were observed [15]. The mice treated with HBS or ExoSpHL presented with tumors affected by necrosis only in the central region (Figure 5A). On the other hand, the animals treated with DOX, SpHL-DOX, or ExoSpHL-DOX presented with extensive necrosis and few areas of viable cells due to the DOX-induced cell death (Figure 5B).

**Figure 6:**
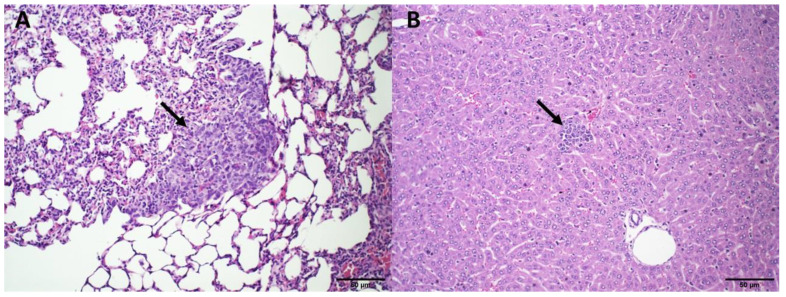
Representative photomicrographs of metastatic foci in the lungs (**A**) and liver (**B**) of female Balb/c mice bearing 4T1 breast tumors treated with HBS (control), ExoSpHL, or DOX at a cumulative dose of 25 mg/kg, SpHL-DOX at a cumulative dose of 25 mg/kg, or ExoSpHL-DOX at a cumulative dose of 25 mg/kg. Arrows indicate tumor metastasis. HE, scale bar = 50 µm.

The 4T1 murine breast cancer is highly tumorigenic and invasive, wherein metastatic foci are observed in various organs [15]. The lungs and liver are common organs affected by the appearance of 4T1 tumor metastases. Pulmonary histology revealed metastatic foci in the animals in all the treatment groups (Figure 6A). The main difference was related to the number of animals with pulmonary metastasis. In all the animals treated with HBS, metastatic foci were observed. However, in the animals treated with ExoSpHL, free DOX, SpHL-DOX, or ExoSpHL-DOX, few metastatic foci were observed. Additionally, the ExoSpHL-DOX-treated group exhibited fewer metastatic foci in a semi-quantitative comparison with the SpHL-DOX and free DOX treatments (Table 4). Multiple metastatic foci in the liver were observed in the animals treated with HBS or ExoSpHL, with no difference between them. Meanwhile, few metastatic foci were observed in the animals treated with DOX, SpHL-DOX, and ExoSpHL-DOX. However, in the liver, there was no difference between the groups treated with DOX, SpHL-DOX, and ExoSpHL-DOX (Figure 6B).

**Figure 7:**
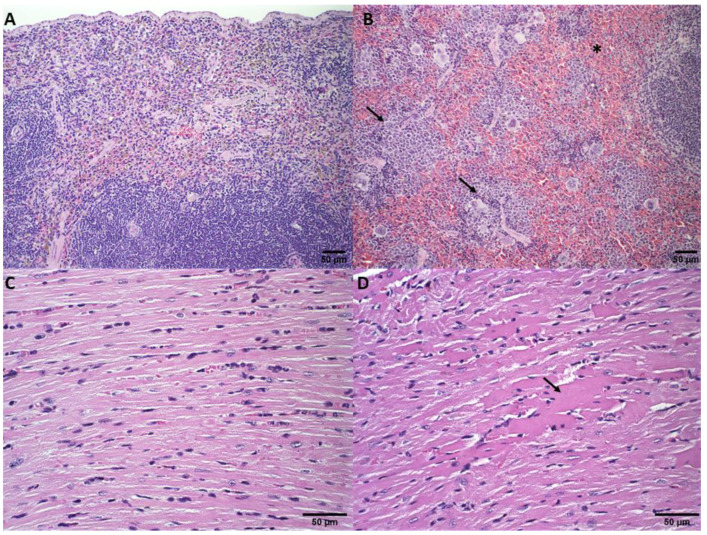
Histological sections of the spleen (**A**,**B**) and heart (**C**,**D**) of female Balb/c mice bearing 4T1 breast tumors. (**A**) Represents the groups treated with SpHL-DOX and ExoSpHL-DOX. Normal spleen. (**B**) Represents the groups treated with HBS, ExoSpHL, and DOX. The asterisk indicates red pulp hyperplasia, and arrows indicate the white pulp hyperplasia of the spleen. (**C**) Represents the groups treated with HBS and ExoSpHL. Normal heart. (**D**) Represents the groups treated with DOX, SpHL-DOX, and ExoSpHL-DOX. The arrow indicates the areas of degenerative hyalinization in the heart. HE, scale bar = 50 µm.

The macroscopic analysis of the spleen showed splenomegaly in the animals treated with HBS or ExoSpHL, which is commonly observed in mice with 4T1 murine breast cancer. In the DOX, SpHL-DOX, and ExoSpHL-DOX treatments groups, the spleen size was normal, indicating that the treatments were able to reverse the splenomegaly. Regarding the histological analysis, the spleen tissue of the animals treated with SpHL-DOX or ExoSpHL-DOX was preserved (Figure 7A). The spleens of the mice treated with HBS, ExoSpHL, or DOX showed white and red pulp hyperplasia (Figure 7B). The cardiac muscle analysis revealed that, compared to the control (Figure 7C), after treatments with free DOX, SpHL-DOX, and ExoSpHL-DOX, there were focal areas of degenerative hyalinization (Figure 7D). Regarding the extent of the lesions, there was no significant difference, and the pattern was similar to that found in the acute toxicity study. Histological sections of all the groups and organs analyzed that showed some difference compared to the control are presented in Appendix C.

## 4. Discussion

Tumor-cell-derived exosomes contain proteins and lipids similar to the cells that secrete them and fuse preferentially with their cells of origin, allowing for a higher concentration of drug delivery in the tumor [16]. Exosome–liposome hybrid nanocarriers have been developed to enhance drug delivery, improve the treatment of metastatic cancer, and overcome chemoresistance in cancer [16,17,18,19]. Based on these findings, our research group developed a hybrid nanocarrier of tumor-derived exosomes fused with long-circulating and pH-sensitive liposomes containing DOX (ExoSpHL-DOX) for the treatment of breast cancer. Our results showed that the developed formulation was stable for 60 days and presented a high DOX encapsulation percentage and a DOX release that was pH-dependent on the medium. Furthermore, our data showed the cytotoxic potential of ExoSpHL-DOX against 4T1 breast cancer cells [7]. In this study, we investigated the acute toxicity and antitumor efficacy of ExoSpHL-DOX. The data obtained indicated that the LD50 for the free DOX treatment is between 12.5 and 15 mg/kg. Meanwhile, for the SpHL-DOX and ExoSpHL-DOX treatments, the LD50 is between 17.5 and 20 mg/kg. As expected, the LD50 values were higher when DOX encapsulated in liposomes or the exosome–liposome hybrid nanocarrier was administered compared to the free form. Similar results were previously found by our research group, where female Balb/c mice treated with long-circulating and pH-sensitive liposomes containing DOX (SpHL-DOX) at a dose of 15 mg/kg showed a decrease in the morbidity and reduced renal, hepatic, and cardiac toxicity of DOX when compared to free DOX at a dose of 15 mg/kg [12]. Another study of acute toxicity of long-circulating and pH-sensitive liposomes containing paclitaxel (PTX):DOX at a molar ratio of 1:10 administered to female Balb/c mice revealed an LD50 value between 28.9 and 34.7 mg/kg, while the free PTX:DOX treatment at a molar ratio of 1:10 presented an LD50 between 20.8 and 23.1 mg/kg [20]. Even though the three animals died after treatment with SpHL-DOX or ExoSpHL-DOX at a dose of 20 mg/kg, it is worth mentioning that the treatment with SpHL-DOX caused twice as much weight loss as ExoSpHL-DOX. The hematological analysis showed leukocytosis and anemia in the animals treated with free DOX at a dose of 12.5 mg/kg. Anemia is a frequent alteration that presents after chemotherapy treatment [21]. However, the increase in the white blood cells was not expected, since after DOX administration, leukopenia is common [12]. In contrast, no changes were observed after treatment with both doses of SpHL-DOX and ExoSpHL-DOX, indicating no signs of toxicity in the hematological parameters. The biochemical analyses revealed the renal toxicity of the free DOX treatment at a dose of 12.5 mg/kg, with an increase in the plasmatic urea levels. Renal damage was also confirmed by histopathology, which showed tubule dilation and hyalinization of the glomeruli after treatment with both doses of free DOX and SpHL-DOX at a dose of 17.5 mg/kg. On the other hand, animals treated with both doses of ExoSpHL-DOX showed no signs of nephrotoxicity, indicating that the presence of exosomes may alter the distribution of DOX and reduce kidney damage, even at the highest dose of the drug [22]. As an indicator of cardiac injury, the CK-MB level was found to be increased after the treatments with free DOX at a dose of 12.5 mg/kg and SpHL-DOX and ExoSpHL-DOX at a dose of 17.5 mg/kg. The increase in the CK-MB value was greater following the free DOX treatment at a dose of 12.5 mg/kg than that induced by the liposomal and exosome–liposomal formulations at the highest dose. Cardiac damage due to the free DOX treatment was also confirmed by multifocal areas of cardiomyocyte vacuolization observed in the histopathology of the animals treated with both doses of free DOX (10 and 12.5 mg/kg). However, discrete cardiomyocyte vacuolization was observed for both doses of SpHL-DOX and ExoSpHL-DOX. These findings are in agreement with a previous study performed by our research group, where female Balb/c mice were treated with free DOX at doses of 10 and 15 mg/kg and the SpHL-DOX treatment at doses of 10 and 15 mg/kg [12], and they can be explained by the lower accumulation of liposomes and exosomes in the heart, due to juxtaposed blood vessels and well-developed lymphatic system [22,23]. In this study, acute toxicity in the spleen and liver was also investigated. Due to the known uptake of liposomes by the liver and spleen, the monitoring of the toxicity of these organs is very important [24]. Splenic toxicity was observed by histopathology only in the groups treated with free DOX. The histological analysis revealed liver damage in the animals treated with both doses of free DOX and liposomal and exosome–liposomal formulations at a dose of 17.5 mg/kg. However, there was no change in the plasma levels of ALT and AST for all the treatments. The antitumor efficacy of the ExoSpHL-DOX treatment was evaluated in the Balb/c female mice with 4T1 breast tumors. The blank hybrid nanocarrier of tumor-derived exosomes fused with long-circulating and pH-sensitive liposomes did not inhibit the tumor growth and showed no signs of toxicity, as was also the case for the HBS treatment. From the obtained results of the tumor volume growth and inhibition of the tumor volume growth, it was observed that the three treatments with DOX showed a higher antitumor effect compared to the control group. However, in terms of efficacy, there was no superiority of the hybrid nanocarrier of the tumor-derived exosomes fused with long-circulating and pH-sensitive liposomes compared to free DOX. Regarding the body weight of the animals, the three treatments containing DOX caused the same body weight loss, similar to another study performed by our research group, in which female Balb/c mice received treatments of free DOX, SpHL-DOX, and long-circulating and pH-sensitive folate-coated liposomes containing DOX (SpHL-DOX-Fol) at a cumulative dose of 20 mg/kg [9]. To verify the antimetastatic activity of the ExoSpHL-DOX treatment, the organs commonly exhibiting the appearance of 4T1 tumor metastases were analyzed by histopathology. The hybrid nanocarrier of the tumor-derived exosomes fused with long-circulating and pH-sensitive liposomes reduced the number of metastatic foci in the lungs, even when it did not contain DOX. Regarding liver metastasis, few metastatic foci were found after the treatments with the formulations containing DOX, and treatment with the blank hybrid nanocarrier of the tumor-derived exosomes fused with long-circulating and pH-sensitive liposomes did not inhibit the liver metastasis. Recent works in the literature have shown the inhibition of metastasis after exosome treatment, but the reasons are still being explored. The innate organotropism capacity of exosomes, the capture and neutralization of circulating tumor cells, and the miRNA content of exosomes are probably involved in this ability to inhibit metastasis [25,26]. Therefore, the hybrid nanocarrier of tumor-derived exosomes fused with long-circulating and pH-sensitive liposomes could serve as a promising nanocarrier for the inhibition of breast cancer metastases, mainly in the lungs.

## 5. Conclusions

In conclusion, the results of the present study demonstrated that the toxicity of the ExoSpHL-DOX treatment is lower than the free DOX treatment, proving that exosome–liposome hybrid nanocarriers are capable of delivering higher doses of DOX without causing serious organ and tissue damage and with reduced adverse effects. In terms of the antitumor efficacy, the ExoSpHL-DOX treatment showed a higher antitumor effect compared to the control. Furthermore, ExoSpHL-DOX reduced the number of metastatic foci in the lungs, even when the exosome–liposome hybrid nanocarrier did not contain DOX. These results indicate that ExoSpHL-DOX may be a promising nanocarrier for the treatment of breast cancer, reducing toxicity and inhibiting metastasis, mainly in the lungs.

## 6. Patents

Oliveira, M.C.; Gomes, E.R.; Sabino, A.P. “Composição farmacêutica contendo doxorrubicina encapsulada em vesículas híbridas de exossomas tumorais e lipossomas pH-sensíveis, processo e uso”. Instituto Nacional da Propriedade Industrial, nr. BR 10 2021 024659 6 (2021).

## Figures and Tables

**Table 1:** Hematological parameters for healthy Balb/c mice treated with different doses of free DOX, SpHL-DOX, or ExoSpHL-DOX.

Blood Components	Control	Free DOX		SpHL-DOX		ExoSpHL-DOX	
		10 mg/kg	12.5 mg/kg	15 mg/kg	17.5 mg/kg	15 mg/kg	17.5 mg/kg
WBC (10^3^/mm^3^)	4.95 ± 1.13	4.55 ± 1.62 ^b^	9.53 ± 2.12 ^a^	4.20 ± 1.18 ^b^	4.85 ± 0.41 ^b^	5.83 ± 1.40 ^b^	4.48 ± 0.82 ^b^
AGRANULOCYTES (10^3^/mm^3^)	3.68 ± 0.99	3.15 ± 1.25 ^b^	7.15 ± 2.58 ^a^	2.58 ± 0.95 ^b^	2.87 ± 0.30 ^b^	3.77 ± 0.97 ^b^	3.00 ± 0.60 ^b^
GRANULOCYTES (10^3^/mm^3^)	1.27 ± 0.27	1.40 ± 0.41 ^b^	2.80 ± 1.05 ^a^	1.34 ± 0.46 ^b^	1.98 ± 0.36 ^b^	2.07 ± 0.59 ^b^	1.48 ± 0.26 ^b^
RBC (10^6^/mm^3^)	6.26 ± 0.72	5.95 ± 0.45 ^b^	4.18 ± 0.36 ^a^	6.06 ± 0.25 ^b^	5.79 ± 0.18 ^b^	5.30 ± 0.26 ^b^	6.10 ± 0.14 ^b^
HGB (g/dL)	12.68 ± 2.26	11.43 ± 1.06 ^b^	8.40 ± 1.35 ^a^	11.72 ± 0.64 ^b^	11.53 ± 0.45 ^b^	10.23 ± 0.43	12.10 ± 0.41 ^b^
HCT (%)	30.90 ± 3.47	30.00 ± 2.05 ^b^	21.55 ± 1.52 ^a^	29.68 ± 1.30 ^b^	28.87 ± 1.01 ^b^	26.82 ± 2.03 ^b^	30.04 ± 0.86 ^b^
PLT (10^3^/mm^3^)	338.20 ± 22.66	254.2 ± 24.70	335.50 ± 78.57	351.80 ± 57.90	333.80 ± 61.90	314.20 ± 40.51	314.00 ± 79.53

WBC: white blood cells; RBC: red blood cells; HGB: hemoglobin; HCT: hematocrit; PLT: platelet. The results are presented as mean ± standard deviation from the mean (*n* = 6, except for free DOX treatment at a dose of 12.5 mg/kg *n* = 5). ^a^ Statistical significance compared to control (HBS) (*p* < 0.05). ^b^ Statistical significance compared to free DOX treatment at a dose of 12.5 mg/kg (*p* < 0.05). Data were evaluated by one-way ANOVA (Tukey’s post-test). If the data were abnormal, the Kruskal–Wallis test with Dunn’s post-test was used.

**Table 2:** Biochemical parameters for healthy Balb/c mice treated with different doses of free DOX, SpHL-DOX, or ExoSpHL-DOX.

Biochemical Parameters	Control	Free DOX		SpHL-DOX		ExoSpHL-DOX	
		10 mg/kg	12.5 mg/kg	15 mg/kg	17.5 mg/kg	15 mg/kg	17.5 mg/kg
Creatinine (mg/dL)	0.30 ± 0.09	0.22 ± 0.07	0.29 ± 0.12	0.19 ± 0.04	0.20 ± 0.03	0.31 ± 0.06	0.22 ± 0.04
Urea (mg/dL)	35.54 ± 3.92	40.51 ± 18.06 ^b^	149.10 ± 14.89 ^a^	34.82 ± 9.91 ^b^	39.20 ± 13.33 ^b^	31.16 ± 1.73 ^b^	30.63 ± 1.37 ^b^
ALT (U/L)	44.09 ± 7.34	52.97 ± 15.68	48.01 ± 9.34	45.54 ± 11.21	56.39 ± 10.01	47.72 ± 5.86	51.67 ± 7.71
AST (U/L)	124.30 ± 24.72	93.78 ± 25.79	127.80 ± 10.44	109.20 ± 41.60	128.00 ± 26.19	110.70 ± 30.26	126.30 ± 24.95
CK-MB (U/L)	28.31 ± 6.36	33.46 ± 13.55 ^b,c^	89.34 ± 8.03 ^a^	33.90 ± 10.33 ^b^	52.49 ± 13.51 ^a,b^	25.99 ± 7.47 ^b,c,d^	49.11 ± 6.58 ^a,b^

The results are presented as mean ± standard deviation from the mean (*n* = 6, except for free DOX treatment at a dose of 12.5 mg/kg *n* = 5). ^a^ Statistical significance compared to control group (HBS) (*p* < 0.05). ^b^ Statistical significance compared to free DOX treatment at a dose of 12.5 mg/kg (*p* < 0.05). ^c^ Statistical significance compared to SpHL-DOX treatment at a dose of 17.5 mg/kg (*p* < 0.05). ^d^ Statistical significance compared to ExoSpHL-DOX treatment at a dose of 17.5 mg/kg (*p* < 0.05). Data were evaluated by one-way ANOVA (Tukey’s post-test). If they were abnormal, the Kruskal–Wallis test with Dunn’s post-test was used.

**Table 3:** Relative tumor volume and tumor growth inhibition after the administration of HBS, ExoSpHL, free DOX, SpHL-DOX, and ExoSpHL-DOX by the intravenous route.

Treatment	RTV	TGI (%)
HBS (control)	6.1 ± 0.9	-
ExoSpHL	6.1 ± 0.6	-
Free DOX 5 mg/kg	2.9 ± 0.4 ^a^	52.5
SpHL-DOX 5 mg/kg	2.8 ± 0.1 ^a^	54.1
ExoSpHL-DOX 5 mg/kg	2.7 ± 0.4 ^a^	55.7

The results are presented as mean ± standard error (*n* = 6). ^a^ Statistical significance compared to the control (HBS) and ExoSpHL (*p* < 0.05).

**Table 4:** Number of metastatic foci in the lungs of female Balb/c mice bearing 4T1 breast tumors treated with HBS, ExoSpHL, free DOX, SpHL-DOX, or ExoSpHL-DOX. Animals received each treatment intravenously five times at a dose of 5 mg/kg every 2 days.

		HBS	ExoSpHL	DOX	SpHL-DOX	ExoSpHL-DOX
	Animal 1	+	+	0	+	0
	Animal 2	++	0	++	0	0
Score	Animal 3	+	+	+	+	+
	Animal 4	++	0	0	0	0
	Animal 5	++	0	0	+	0

Data are expressed as scores: 0, no metastasis detected; +, 1–3 metastatic foci; ++, 4–7 metastatic foci.

**Figure A3:**
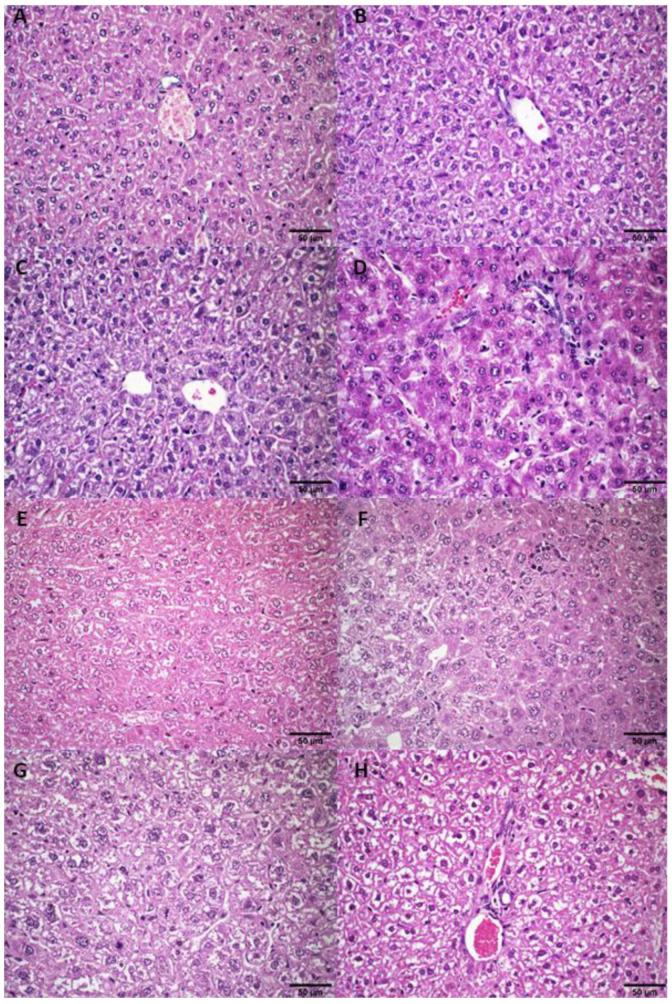
Histological sections of female Balb/c mice liver. (**A**) Control group (HBS), (**B**) ExoSpHL, and (**C**) SpHL-DOX at a dose of 15 mg/kg, (**D**) ExoSpHL-DOX at a dose of 15 mg/kg, (**E**) DOX at a dose of 10 mg/kg, (**F**) DOX at a dose of 12.5 mg/kg, (**G**) SpHL-DOX at a dose of 17.5 mg/kg, (**H**) ExoSpHL-DOX at a dose of 17.5 mg/kg.

**Figure A4:**
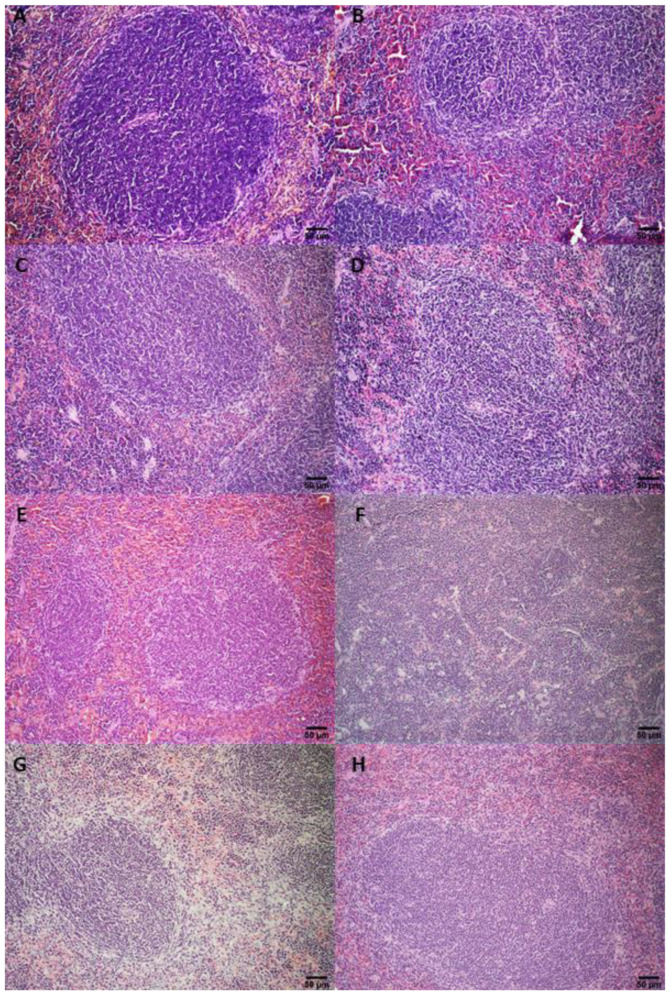
Histological sections of female Balb/c mice spleen. (**A**) Control group (HBS), (**B**) ExoSpHL, and (**C**) SpHL-DOX at a dose of 15 mg/kg, (**D**) SpHL-DOX at a dose of 17.5 mg/kg, (**E**) ExoSpHL-DOX at a dose of 15 mg/kg, (**F**) ExoSpHL-DOX at a dose of 17.5 mg/kg, (**G**) DOX at a dose of 10 mg/kg, (**H**) DOX at a dose of 12.5 mg/kg.

**Figure A5:**
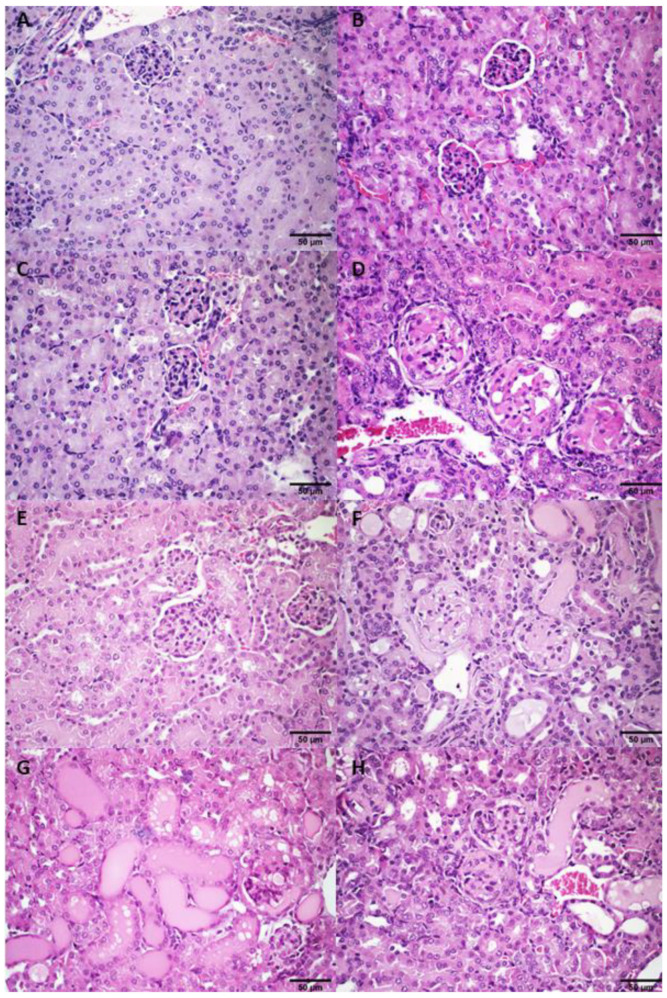
Histological sections of female Balb/c mice kidney. (**A**) Control group (HBS), (**B**) ExoSpHL, and (**C**) SpHL-DOX at a dose of 15 mg/kg, (**D**) ExoSpHL-DOX at a dose of 15 mg/kg, (**E**) ExoSpHL-DOX at a dose of 17.5 mg/kg, (**F**) DOX at a dose of 10 mg/kg, (**G**) DOX at a dose of 12.5 mg/kg, (**H**) SpHL-DOX at a dose of 17.5 mg/kg.

**Figure A6:**
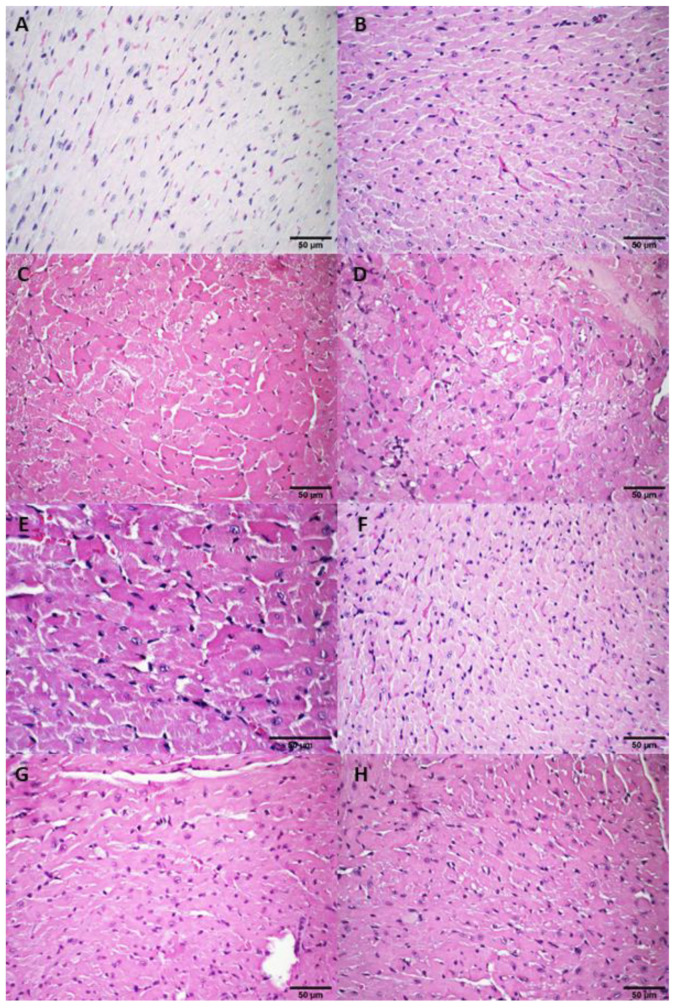
Histological sections of female Balb/c mice heart. (**A**) Control group (HBS), (**B**) ExoSpHL, and (**C**) DOX at a dose of 10 mg/kg, (**D**) DOX at a dose of 12.5 mg/kg, (**E**) SpHL-DOX at a dose of 15 mg/kg, (**F**) ExoSpHL-DOX at a dose of 15 mg/kg, (**G**) SpHL-DOX at a dose of 17.5 mg/kg, (**H**) ExoSpHL-DOX at a dose of 17.5 mg/kg.

**Figure A7:**
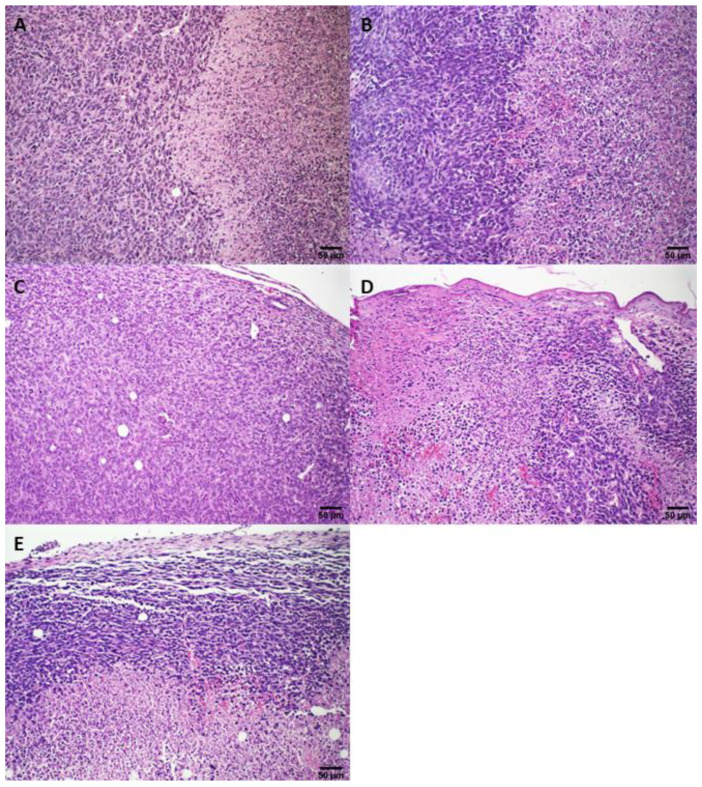
Histological sections of primary tumor of female Balb/c mice bearing 4T1 breast tumors treated with (**A**) control group (HBS), (**B**) ExoSpHL, or (**C**) DOX at a cumulative dose of 25 mg/kg, (**D**) SpHL-DOX at a cumulative dose of 25 mg/kg, (**E**) ExoSpHL-DOX at a cumulative dose of 25 mg/kg.

**Figure A8:**
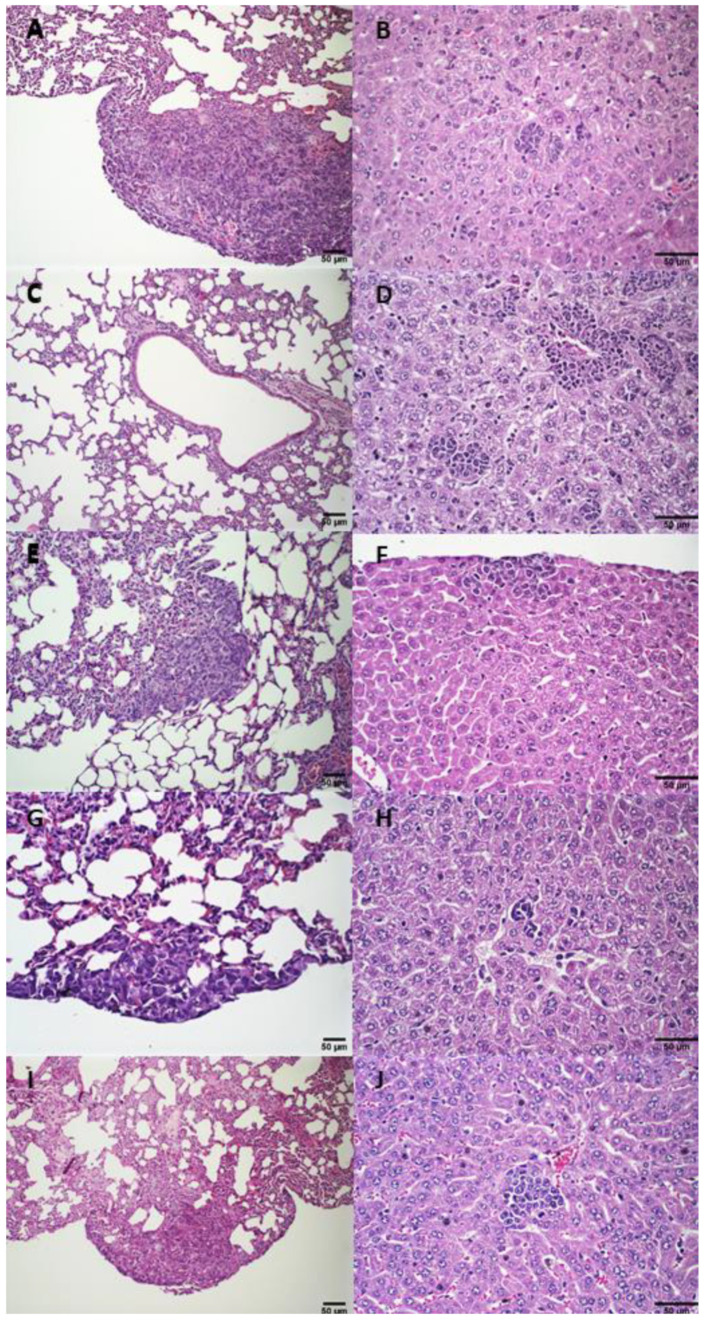
Histological sections of metastatic foci in lungs and liver of female Balb/c mice bearing 4T1 breast tumors treated with HBS (**A**,**B**), ExoSpHL (**C**,**D**), and DOX at a cumulative dose of 25 mg/kg (**E**,**F**), SpHL-DOX at a cumulative dose of 25 mg/kg (**G**,**H**), and ExoSpHL-DOX at a cumulative dose of 25 mg/kg (**I**,**J**).

**Figure A9:**
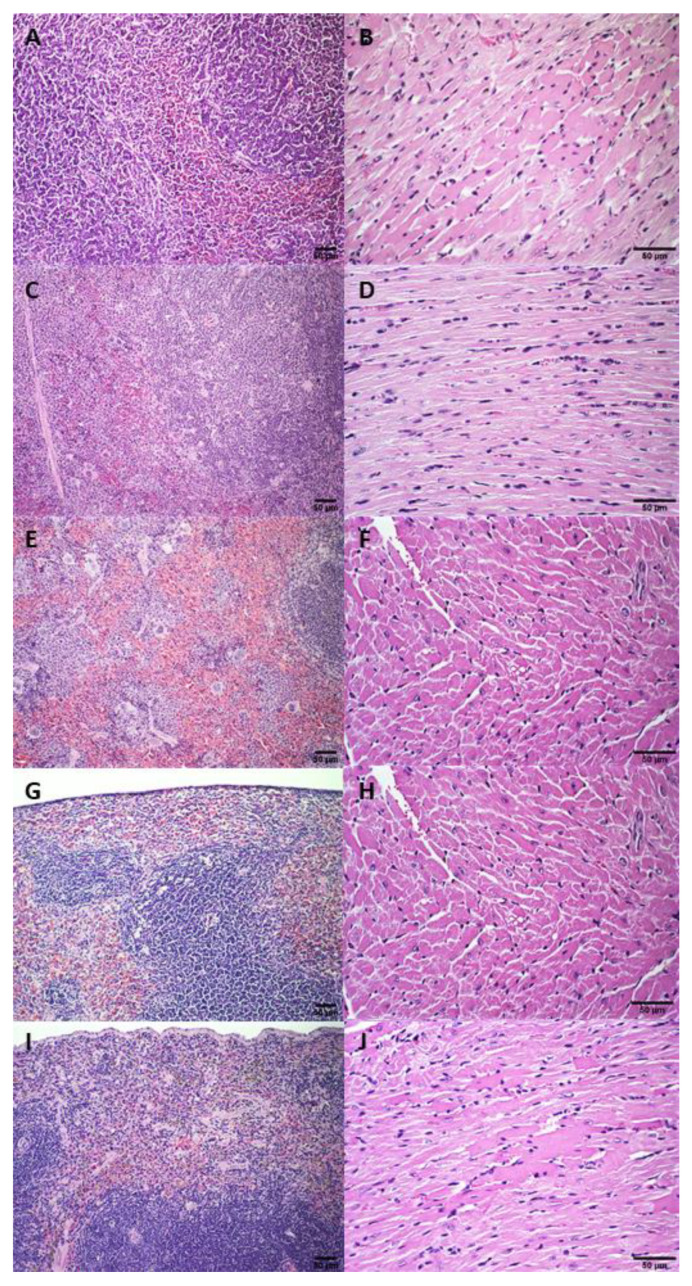
Histological sections of the spleen and heart of female Balb/c mice bearing 4T1 breast tumors treated with HBS (**A**,**B**), ExoSpHL (**C**,**D**), and DOX at a cumulative dose of 25 mg/kg (**E**,**F**), SpHL-DOX at a cumulative dose of 25 mg/kg (**G**,**H**), and ExoSpHL-DOX at a cumulative dose of 25 mg/kg (**I**,**J**).
